# Challenges in Developing Evidence-Based Recommendations Using the GRADE Approach: The Case of Mental, Neurological, and Substance Use Disorders

**DOI:** 10.1371/journal.pmed.1000322

**Published:** 2010-08-31

**Authors:** Corrado Barbui, Tarun Dua, Mark van Ommeren, M. Taghi Yasamy, Alexandra Fleischmann, Nicolas Clark, Graham Thornicroft, Suzanne Hill, Shekhar Saxena

**Affiliations:** 1 Department of Medicine and Public Health, Section of Psychiatry and Clinical Psychology, University of Verona, Verona, Italy; 2 Department of Mental Health and Substance Abuse, World Health Organization, Geneva, Switzerland; 3 Health Services Research Department, Institute of Psychiatry, King's College, London, United Kingdom; 4 Essential Medicines and Pharmaceutical Policies, World Health Organization, Geneva, Switzerland

## Abstract

Corrado Barbui and colleagues describe their use and adaptation of the GRADE approach in developing the guidelines for the WHO mental health Gap Action Programme (mhGAP).

## Introduction

The World Health Organization (WHO) has been criticized recently for not consistently making recommendations based on systematic reviews of the best available evidence and for the quality of some of its guidelines [1]–[3]. In 2007, WHO put in place procedures for developing transparent, evidence-based guidelines based on the Grading of Recommendations Assessment, Development and Evaluation (GRADE) methodology [4],[5]. This methodology, developed by an international network of methodologists with an interest in grading quality of evidence and strength of recommendations (Box 1), has now been used to produce WHO guidelines for several topics. These include rapid advice guidelines for the pharmacological management of human H5N1 virus infection [6],[7] and guidelines on a single specific clinical topic such as psychosocially assisted pharmacological treatment of opioid dependence [8]. However, the GRADE approach has not yet been applied to develop recommendations that cover a broad range of conditions and interventions.

**Box 1. Main Features of the GRADE Methodology**

GRADE is an approach for creating clinical practice guidelines based on an explicit assessment of the evidence base.

GRADE is not a system for performing systematic reviews and meta-analyses (it is not a systematic review tool as, for example, the RevMan software of the Cochrane Collaboration at http://www.cc-ims.net/revman).

The GRADE approach is suitable for (a) summarizing the evidence extracted from systematic reviews and meta-analyses into “Summary of Findings (SoF) tables”; (b) grading the quality of evidence summarized in SoF tables; and (c) grading the strength of treatment recommendations.

GRADE separates the judgment on quality of evidence from strength of recommendations.

An application called GRADE Profiler (GRADEpro) has been developed to summarize the evidence and grade its quality. GRADEpro can be freely downloaded at http://www.gradeworkinggroup.org/toolbox/index.htm.

Additional information on the GRADE methodology and on the GRADE working group can be found at http://www.gradeworkinggroup.org/index.htm.

WHO is in the process of developing a model intervention guide within its mental health Gap Action Programme (mhGAP) [9]. The model intervention guide provides recommendations to facilitate care at first and second level facilities by the non-specialist health care providers in low- and middle-income countries (Box 2). These recommendations will be based on the GRADE approach. To our knowledge, this is the first exercise involving a systematic evaluation of evidence in this area. Other initiatives, for example the recently published reviews of evidence for packages of care for mental, neurological, and substance use disorders in low- and middle-income countries, did not use GRADE methodology [10]. This paper describes the use and adaptation of the GRADE approach in developing the guidelines for the mhGAP model intervention guide.

**Box 2. Rationale for Developing WHO Recommendations for Mental, Neurological, and Substance Use Disorders**

Mental, neurological, and substance use (MNS) disorders are prevalent throughout the world and are major contributors to morbidity and premature mortality. The treatment gap for these disorders is more than 75% in many low- and lower-middle income countries. Substantial initiatives have been made by in the last decade to bring mental health onto public health agenda [24]–[26], however the task is far from complete. To address this challenge, WHO launched the mental health Gap Action Programme (mhGAP) to scale up mental health services, especially in low- and middle-income countries (LAMIC) [9]. An essential component of mhGAP is to develop a model intervention guide for MNS disorders identified as conditions of high priority for LAMIC. The priority conditions were identified on the basis of high mortality and morbidity, high economic costs, or association with violation of human rights within the area of MNS disorders. These are depression, schizophrenia and other psychotic disorders (including bipolar disorder), suicide prevention, epilepsy, dementia, disorders due to use of alcohol and illicit drugs, and mental disorders in children. Recommendations (i.e., guidelines) on interventions for the management of such high priority conditions form the basis of the mhGAP model intervention guide. Interventions are targeted to health care providers working at a first and second level facility in a health center at a peripheral level or at district level. The first and second level facility includes the basic outpatient and inpatient services provided at these levels. The health care providers could be doctors, nurses, or other cadre of health workers.

## Overview of the Process

**Figure 1:**
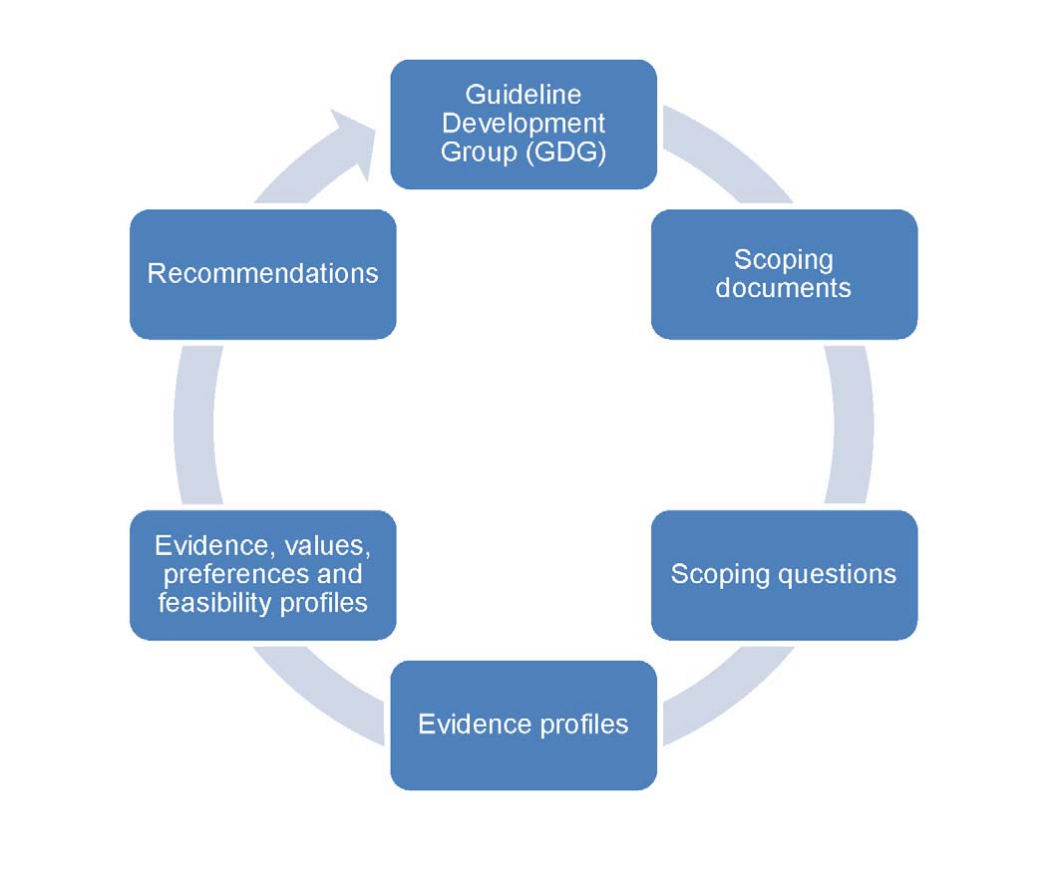
Pathway describing the process of recommendation development. The Guideline Development Group (GDG) developed the scoping documents and the scoping questions. For each scoping question, a WHO working group drafted evidence profiles and profiles incorporating values, preferences, and feasibility considerations. Recommendations were subsequently drafted and submitted to the GDG for review and approval.

The pathway describing the process of recommendation development is presented in Figure 1. A network of experts were identified to convene the Guideline Development Group (GDG), taking into consideration multidisciplinary expertise and adequate regional and gender representation. The multidisciplinary expertise included guideline development methodology, mental health, neurology, substance use, primary care, public health, epidemiology, and policy making. A review of potential conflicts of interest was carried out in agreement with the WHO handbook [5]. The GDG developed scoping questions and defined outcomes. For each scoping question, evidence was aggregated and synthesized following the GRADE methodology. The evidence profiles were then supplemented by noting values, preferences, and feasibility considerations. Recommendations were subsequently drafted and submitted to the GDG for review, modification, and approval.

We report here some challenges encountered during the process of evidence review and developing recommendations. Developing recommendations in the context of these disorders is complex, as evidence directly from low- and middle-income settings is still limited, and values, preferences, and feasibility issues tend to play a large role in informing recommendations.

## Challenges: From Scoping Questions to Evidence Profiles

### Formulating Questions and Rating Outcomes

**Figure 2:**
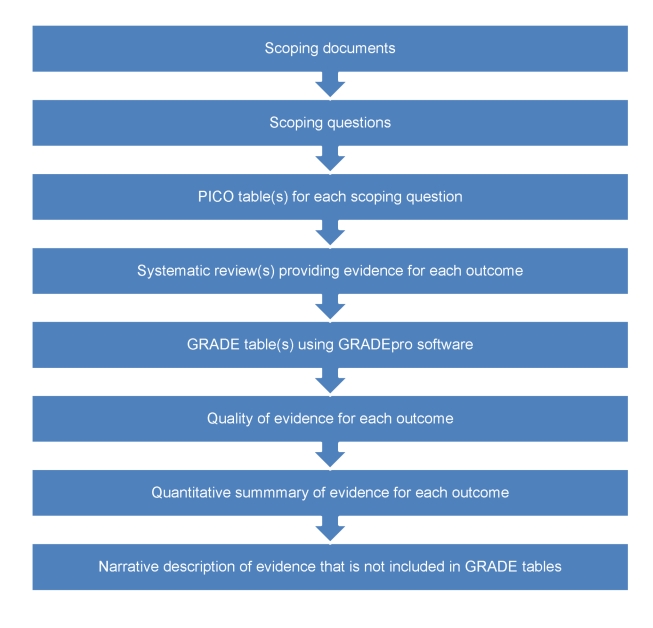
From scoping questions to evidence profiles: flow-chart describing the process to identify, summarize, and rate the evidence for each scoping question.

The pathway from defining scoping questions to developing evidence profiles is described in Figure 2. Scoping questions were formulated using the PICO framework (Population, Intervention, Comparator, Outcome) [11], which was useful for translating narrative questions into a format suitable for searching and synthesizing evidence. The conditions considered of high priority for low- and middle-income countries reported in Box 2 were the focus of scoping questions. In addition to scoping questions, outcomes critical or important for evidence review and decision making and recommendations were agreed upon by the GDG. A key challenge was that there was an enormous number of potential questions, which would require years to answer. Thus, when formulating and selecting scoping questions, GDG members were asked to identify the most important questions, including any areas of uncertainty or controversy or where changes in policy or practice was needed. For example, given that in some countries the proportion of individuals with epilepsy who do not receive adequate drug treatment is high, with controversy related to side effects of anti-epileptic drugs, one scoping question focused on the beneficial and harmful consequences of these drugs. We note that getting the scoping right is critical to the overall process of making recommendations.

Identification of critical and important outcomes was a crucial process, as outcomes guided the subsequent phases of evidence retrieval, synthesis, and making recommendations. Based on the GRADE methodology, outcomes were grouped into three categories: critical, important, and not important. Very often, clinical trials report those outcomes (e.g., symptoms) that can be easily measured, while omitting other key outcomes (e.g., human rights violations) that are more difficult to quantify. During the outcome rating process, the GDG members explicitly considered the value of choosing and rating outcomes that should be measured rather than those that have been measured.

### Selecting the Measure That Best Describes Each Outcome

For each scoping question, the experts and WHO focal points formed review teams that as a first step searched for systematic reviews from databases and existing evidence-based guidelines that have tried to answer a similar scoping question. In case no synthesized evidence was available, new systematic reviews were commissioned. The review teams summarized the evidence base using the GRADE profiler software, a tool developed to help transparently summarize the evidence and grade its quality (see Box 1). The GRADE process required us to summarize evidence and grade its quality for each of the outcomes, using systematic reviews and meta-analyses. It often happened that, for each outcome, more than one measure was available in the selected systematic review. This introduced potential inconsistency and bias because no explicit criteria were available to guide the selection of the measure for one outcome. This issue is especially challenging in the field of mental health, as outcomes are typically measured by means of rating scales, and very often several rating scales purporting to measure the same concept are included in trials and meta-analyses. Systematic reviews may consequently include four to five measures for the same outcome.

In our process, to increase transparency and consistency between raters, explicit instructions were developed to help reviewers use the same background logic in making the choice (Text S1), although the possibility of adopting a different logic was considered acceptable if justified and reported. We note that the absence of instructions on using GRADE leaves the possibility of inconsistency and bias, because raters may implicitly or explicitly choose the measures with the results that better match their a priori opinions.

### Reproducibility and Consistency

One of the key challenges in evaluating the quality of evidence with GRADE is that some factors (i.e., limitations and indirectness) imply a judgement on the quality of a group of trials, while other factors (i.e., inconsistency, imprecision, publication bias) imply a judgement on the meta-analytic process of data aggregation (see Box 3 for a definition of these terms). This implies that reviewers, either implicitly or explicitly, are asked to make a different reasoning according to the dimension that is considered: for the factor limitation, for example, raters need to judge the risk of bias of each single trial included in the selected systematic review and, secondly, to make a judgment on whether the estimated proportion of trials at risk of bias causes a risk of bias for the entire evidence base. In areas where there are only a few trials this may not be a serious challenge. In areas characterized by the presence of many randomized trials, however, such as antidepressants for major depression where single reviews may include more than 100 trials, this posed a practical problem, in terms of feasibility (to access all primary studies), and also in terms of reproducibility and consistency [12]. For dimensions that refer to the meta-analytic process, only a single judgment is required, but even in this case it is very likely that different raters would apply different criteria, and that the same rater may implicitly apply different criteria in different situations.

**Box 3. Factors to Consider in the Assessment of the Quality of the Evidence according to the GRADE Approach**

Study limitations: Limitations in the study design that may bias the overall estimates of the treatment effect.

Inconsistency: Unexplained differing estimates of the treatment effect (i.e., heterogeneity or variability in results) across studies.

Indirectness: The question being addressed by the guideline panel is different from the available evidence regarding the population, intervention, comparator, or outcome.

Imprecision: Results are imprecise when studies include relatively few patients and few events and thus have wide confidence intervals around the estimate of the effect.

Publication bias: Systematic underestimate or overestimate of the underlying beneficial or harmful effect due to the selective publication (or reporting) of studies.

Problems with reliability in GRADE are particularly problematic when several raters are involved in the development of recommendations, which is often the case for development of guidelines covering a broad range of conditions. We therefore reasoned that raters should follow the same background logic in assessing the evidence and developed pragmatic instructions to guide the process of grading the quality of the evidence (Text S1), to increase the reproducibility of the process and the consistency of judgements. If the instructions were not followed in any specific circumstances, raters were required to record a reason.

We note that our instructions are inevitably based on rules of thumb in many cases, and may not be automatically extrapolated from one guideline to another, because criteria are based on the perspective that the guideline developers assume and are tuned to the relevant clinical area.

### Publication Bias

According to the GRADE methodology, raters should assess the possibility of publication bias. We note that the instruments for making such a judgment are inadequate. We tried to rely on the funnel plot, although its use is controversial [13],[14] and often was not reported in systematic reviews. We thus used the additional pragmatic criterion of checking if the authors of the systematic review included unpublished material, and we tried to make an additional judgment on whether outcome reporting bias might have occurred. Also, in the latter case, instrumentation was a problem: study protocols were generally not available, and the feasibility of checking this aspect in hundreds of clinical trial is very low. Although publication bias is probably one of the most relevant aspects of the whole process of summarizing and grading the quality of evidence, we realize that only weak methods are currently available to detect it.

### Evidence from Non-Randomized Trials

For many clinical questions, more often than not, evidence in the form of randomized trials is not available. Epidemiological studies and qualitative studies cannot easily be described in GRADE tables, and a risk exists of omitting the contribution of non-randomized research in the development of the evidence profiles. For example, the value of asking about suicide ideation of individuals with specific mental disorders has never been investigated through clinical trials, but still there is indirect epidemiological evidence suggesting that asking increases detection with no major drawbacks, and increased detection is associated with increased treatment and better outcomes. Although this issue was addressed by requiring the inclusion of additional evidence in a narrative section of the evidence profiles, this remains a weak aspect of the whole process. Even for observational studies that investigate the beneficial or harmful effects of interventions, grading is rarely straightforward, as systematic reviews of these studies are typically not available, and the meta-analytical process of pooling their results may often be methodologically inappropriate.

We note that the systematic omission of some non-randomized evidence from GRADE inevitably creates an imbalance between low- and high-level quality of evidence, with low value and consideration given to what is kept out from GRADE tables. This may represent a serious bias in the process of guideline development, considering that each research question requires a proper research design, and clinical trials cannot provide an answer to all health-related questions [15].

## Challenges: From Evidence to Recommendations

### Scientific Evidence versus Values, Preferences, and Feasibility Issues

**Figure 3:**
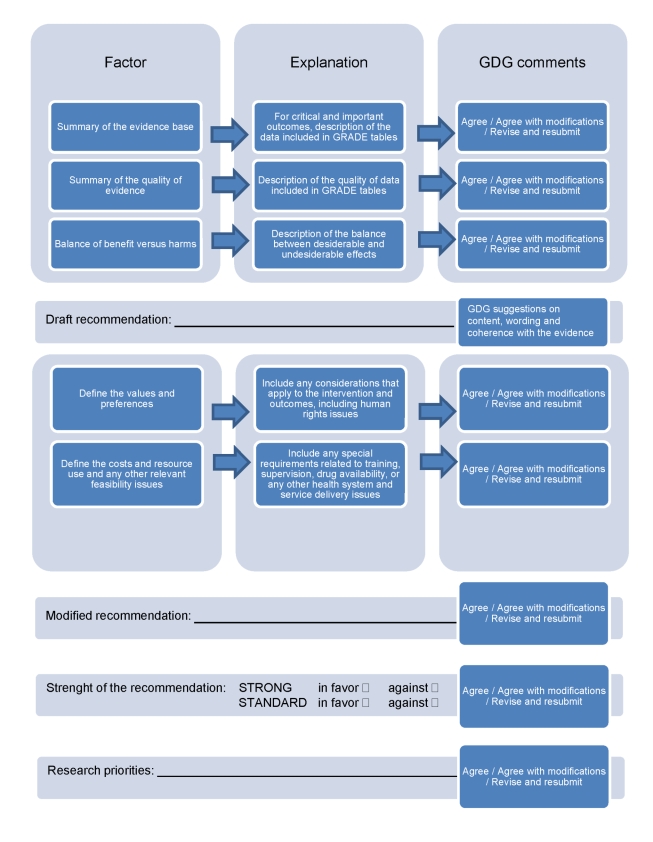
From evidence profiles to recommendations: template describing how evidence, values, preferences, and feasibility issues were considered in making recommendations.

The pathway from aggregating and grading the quality of evidence to developing recommendations is described in Figure 3. While the methodology for developing guidelines is highly developed for aspects related to summarizing and judging the evidence base [16]–[19], the methodology for taking into consideration aspects related to values, preferences, and feasibility issues is much less developed. We have sought to address this imbalance by developing a checklist (Box 4) and a template (Figure 3) that give equal relevance and visibility to both aspects, i.e., the evidence base and the value/preferences and feasibility issues. Firstly, for each scoping question, the review teams summarized the evidence included in the GRADE tables and its quality ratings, and drafted a narrative description of the balance between desiderable and undesiderable effects. This formed the basis for the “zero draft recommendation”. Secondly, the review teams were required to take into consideration values, preferences, and feasibility issues. A checklist of aspects that deserve consideration was developed to increase consistency across scoping questions (Box 4). A modified recommendation, based on the evidence base and on the considerations on values, preferences, and feasibility issues, was then drafted and reported in the table.

**Box 4. Checklist of Feasibility Issues, Values, and Preferences Considered for Each Intervention**

**Feasibility Issues**

- Inclusion in the WHO list of essential medicines and likely availability of medication in LAMIC
- Acquisition cost
- Current treatment skills availability in LAMIC for this intervention
- Specific training requirements (comment if >1.0 day just for this specific intervention)
- Number of sessions, number of minutes per sessions required
- Specific laboratory requirements
- Other equipment requirements
- Continuous supply of medication (comment if sudden disruption of supply could have harmful consequences, e.g., for anti-epileptics)
- Specific supervision requirements (comment if more than one supervisory discussion is needed per 3 months)
- Any other feasibility issues

**Values/Preferences**

- Promotion of social inclusion
- Protection of human rights and dignity (e.g., interventions that are sometimes provided on a non-voluntary basis)
- Prevention of discrimination (and stigma)
- Prevention of medicalization of social problems
- Promotion of individual and family members' capacity and skills
- Any other values

NOTE: Many of these aspects are not absolute concepts, and their relevance may vary according to local context characteristics.

This approach allows for the transparent reporting of how recommendations derived from the scientific evidence may then be modified due to the weight given to other factors (Figure 3). For example, medication treatments backed by robust evidence, such as lithium in the treatment of mood disorders, may not be recommended if regular blood checks, and their interpretation, are not feasible, or if regular drug supply cannot be assured. Similarly, psychological interventions, such as full cognitive behavioral therapy, may not be feasible in many low- and middle-income settings considering the training, supervision, and time needed for this intervention, and that they might need local adaptations to context and culture.

### Recommendations When There Is No or Poor Quality Evidence

For certain key areas, the conventional way of evaluating the quality of evidence revealed no or very poor quality evidence that was not sufficient to make any recommendation. In such instances we relied on the consensus of GDG consisting of international experts, who applied their professional experience and their tacit knowledge. Value-based decisions in mental health are unavoidable, as violation of human rights of people with mental disorders is common. For example, for the strategies aimed at improving community attitudes towards people with mental, neurological, and substance use conditions, the evidence base is very poor and indirect. Nevertheless, because of strong values and the importance of improving community attitudes, the GDG made a recommendation to consider planning and implementation of activities such as anti-stigma campaigns. We note that the added value of GRADE in these circumstances is that it is required to transparently report that some recommendations are based on strong values and weak evidence. The GDG was also requested to make specific statements for recommending research in areas where there is a paucity of evidence (Figure 3).

### Applicability

While the main focus of WHO's mental health work was to develop recommendations for health care providers in low- and middle-income settings, most of the evidence comes from specialist settings of high-income countries. We tried to address this concern as follows. First, the GRADE assessment of directness was used to downgrade the quality of evidence if there were concerns about generalisability of population, intervention comparator, or outcome. Additionally, we included a judgment on the extent to which the characteristics of those who would deliver the intervention in the real world (including context characteristics) match with the characteristics of those who actually delivered the intervention under experimental conditions (e.g., in terms of background education, training, referral possibilities, context). A further aspect was the availability of implementation studies conducted in low- and middle-income countries. For example, the applicability of some brief psychological treatments in low-income settings has been documented in research projects carried out in Uganda and Pakistan [20]–[22], and the results of these practical experiences have been given value in judging the applicability of the intervention and in drafting the recommendations. We realize that despite the fact that these strategies helped mitigate this issue, increasing research in the contexts where the guidelines should eventually be applied is essential to enhance the applicability of research findings into practice.

## Conclusions

Our experience suggests that GRADE may be applied as a useful technical framework for synthesizing and presenting evidence on the effectiveness of clinical interventions. It is a helpful tool to uncover implicit subjectivity, since it requires a systematic, explicit, and judicious approach to interpreting evidence [23]. However, the process may be further improved in the following domains: inclusion of non-randomized evidence and evidence that cannot be meta-summarized and analyzed; better reproducibility and internal consistency; and consideration of the choice of one among several measures for each outcome to reduce the selection bias. Development of recommendations is a complex process that not only involves systematic review and assessment of quality of evidence and balance of benefits and harms, but also explicit consideration of other issues such as value judgments, resource use, and feasibility, which are major considerations. The technicality of GRADE needs to be supplemented by a careful analysis of these additional but essential issues.

## Supporting Information

Text S1 Appendix
(0.30 MB DOC)
