# Supplementary material for: Challenges in Developing Evidence-Based Recommendations Using the GRADE Approach: The Case of Mental, Neurological, and Substance Use Disorders
Source: PLoS Med. 2010 Aug 31;7(8):e1000322. doi: 10.1371/journal.pmed.1000322 (PMC2930877; doi:10.1371/journal.pmed.1000322)
Supplement: Text S1 — Appendix (0.30 MB DOC) [file pmed.1000322.s001.doc]

**Text S1**

**CHOICE OF THE MEASURE THAT BETTER DESCRIBES THE OUTCOME OF INTEREST and PRACTICAL INSTRUCTIONS FOR ASSESSING THE QUALITY OF EVIDENCE INCLUDED IN SYSTEMATIC REVIEWS**

**CHOICE OF THE MEASURE THAT BETTER DESCRIBES THE OUTCOME OF INTEREST**

- Include both dichotomous and continuous outcomes (if possible)

- Select the measure that is considered the “standard” in the disorder under study
- Adapt according to situations (e.g. in child mental health often outcomes are assessed from the parent’s perspective and from the teacher’s perspective) and explain the choice in footnotes (where additional measures may be described to ascertain whether they provide consistent results)

**PRACTICAL INSTRUCTIONS FOR ASSESSING THE QUALITY OF EVIDENCE INCLUDED IN SYSTEMATIC REVIEWS**

**GENERAL PRINCIPLES**

In order to assess the quality of evidence using the GRADE template, it is essential that raters agree on basic criteria to be used to downgrade or upgrade the evidence. This will enhance the consistency and reliability of ratings.

General principles:

(1) A first rater will grade the quality of evidence for each outcome, and will summarize findings using the GRADE template for each outcome. Ratings will be checked for consistency by a second member of the review group. Agreement between raters should be reached (a third rater might be involved in case of disagreement).

(2) According to the GRADE methodology (<http://www.gradeworkinggroup.org/index.htm>) when assessing quality of evidence this diagram should be followed.


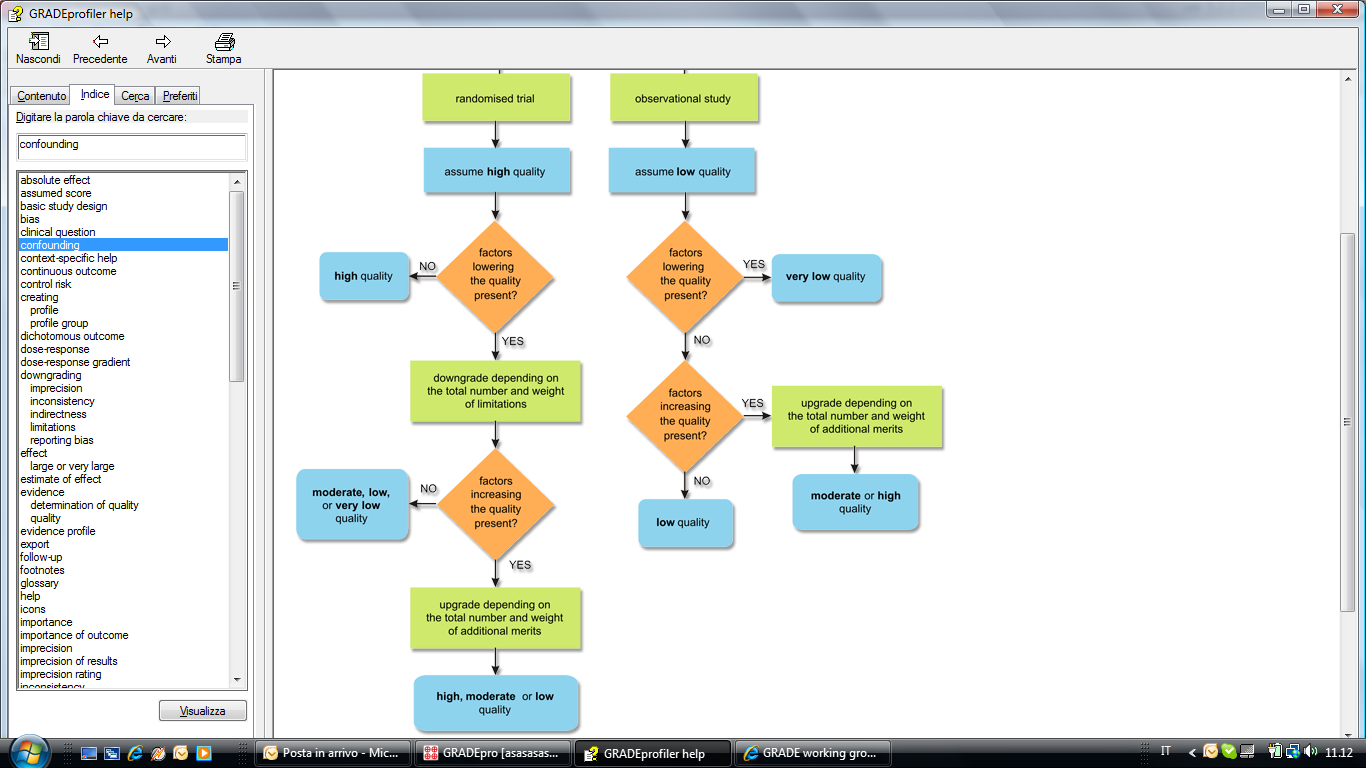


(3) According to the GRADE methodology (<http://www.gradeworkinggroup.org/index.htm>) when assessing the quality of evidence the GRADE general approach should be followed:

- GRADE is not a quantitative system for grading the quality of evidence. Each factor for downgrading or upgrading reflects not discrete categories but **a continuum within each category and among the categories**. When the body of evidence is intermediate with respect to a particular factor, the decision about whether a study falls above or below the threshold for up- or downgrading the quality (by one or more factors) depends on judgment.

- Despite the limitations of breaking continua into categories, treating each criterion for rating quality up or down as discrete categories enhances transparency. Indeed, **the great merit of GRADE is not that it ensures reproducible judgments but that it requires explicit judgment that is made transparent to users.**

NOTE: Observational studies that have been downgraded to very low quality for any reason should not be upgraded.

(4) To achieve transparency and implicity, the GRADE system classifies the quality of evidence in one of four grades (<http://www.gradeworkinggroup.org/index.htm>):

| Grade | Definition |
| --- | --- |
| High | Further research is very unlikely to change our confidence in the estimate of effect. |
| Moderate | Further research is likely to have an important impact on our confidence in the estimate of effect and may change the estimate. |
| Low | Further research is very likely to have an important impact on our confidence in the estimate of effect and is likely to change the estimate. |
| Very low | Any estimate of effect is very uncertain. |

(4) According to the GRADE system, raters are required to make a judgement on studies included in systematic reviews with respect to the following criteria:

(1) LIMITATIONS (RISK OF BIAS)

(2) INCONSISTENCY

(3) INDIRECTNESS

(4) IMPRECISION

(5) REPORTING BIAS

**(1) LIMITATIONS (risk of bias)**

GRADE Definition. Limitations in the study design and implementation may bias the estimates of the treatment effect. Our confidence in the estimate of the effect and in the following recommendation decreases if studies suffer from major limitations. The more serious limitations are, the more likely it is that the quality of evidence will be downgraded. Our confidence in an estimate of effect decreases if studies suffer from major limitations that are likely to result in a biased assessment of the intervention effect. For randomized trials, the following limitations are likely to result in biased results: lack of allocation concealment, lack of blinding, incomplete accounting of patients and outcome events, selective outcome reporting, other (for further details see the GRADEprofiler instructions).

**The following criteria will be followed by WHO raters on LIMITATIONS**:

If one or more of the three criteria reported below is not met in up to 10% of trials included in the systematic review = no downgrading (negligible limitations)

If one or more of the three criteria reported below is not met in 10-30% of trials included in the systematic review = - 1 (serious limitations)

If one or more of the three criteria reported below is not met in more than 30% of trials included in the systematic review = - 2 (very serious limitations)

The three criteria are:

(1) trials are described as randomised;

(2) outcome assessment is described as masked;

(3) dropout rate (both treatment arms) is below or equal to 30% (and dropouts are similarly distributed between treatment arms).

*A different criterion may be followed by WHO raters in exceptional situations. Explanation should be reported as footnote in the corresponding GRADE table.*

**(2) INCONSISTENCY**

GRADE Definition. Inconsistency refers to an unexplained heterogeneity of results across studies. Widely differing estimates of the treatment effect (i.e. heterogeneity or variability in results) across studies suggest true differences in underlying treatment effect. When heterogeneity exists, but investigators fail to identify a plausible explanation, the quality of evidence should be downgraded by one or two levels, depending on the magnitude of the inconsistency in the results (for further details see the GRADEprofiler instructions). Inconsistency may arise from differences in:

- populations (e.g. drugs may have larger relative effects in sicker populations)
- interventions (e.g. larger effects with higher drug doses)
- outcomes (e.g. diminishing treatment effect with time).

Guideline panels or authors of systematic reviews should also consider the extent to which they are uncertain about the underlying effect due to the inconsistency in results and they may downgrade the quality rating by one or even two levels.

**The following criteria will be followed by WHO raters on INCONSISTENCY**:

If visual investigation of forest plots suggests some degree of heterogeneity (supported by a formal test of heterogeneity indicating some degree of heterogeneity, for example I-squared between 50% and 75%) = - 1 (serious inconsistency)

If visual investigation of forest plots suggests high degree of heterogeneity (supported by a formal test of heterogeneity indicating high heterogeneity, for example I-squared higher than 75%) = - 2 (very serious inconsistency)

*A different criterion may be followed by WHO raters in exceptional situations. Explanation should be reported as footnote in the corresponding GRADE table.*

NOTE: Raters will not downgrade for inconsistency when only one study contributes to the evidence base.

**(3) INDIRECTNESS**

GRADE Definition. There are two types of indirectness.

1. Indirect comparison – occurs when a comparisons of intervention A versus B is not available, but A was compared with C and B was compared with C. Such studies allow indirect comparisons of the magnitude of effect of A versus B. Such evidence is of lower quality than head-to-head comparisons of A and B would provide.

2. Indirect population, intervention, comparator, or outcome – the question being addressed by the guideline panel or by the authors of a systematic review is different from the available evidence regarding the population, intervention, comparator, or an outcome.

Indirectness may additionally refer to the extent to which the characteristics of those who will deliver the intervention in the real-world (including context characteristics) match with the characteristics of those who actually delivered the intervention under experimental conditions (in terms of background education, training, referral possibilities, context, other features).

Those making recommendations or authors of systematic reviews should consider the extent to which they are uncertain about the applicability of the evidence to their relevant question and downgrade the quality rating by one or even two levels.

**The following criteria will be followed by WHO raters on INDIRECTNESS**:

The question being addressed by the guideline panel is different from the available evidence regarding the population, intervention, comparator, outcome or regarding the characteristics of those who will deliver the intervention = - 1 (serious doubts about directness)

The question being addressed by the guideline panel is markedly different from the available evidence regarding the population, intervention, comparator, outcome or regarding the characteristics of those who will deliver the intervention = - 2 (very serious doubts about directness)

*A different criterion may be followed by WHO raters in exceptional situations. Explanation should be reported as footnote in the corresponding GRADE table.*

NOTE: If only one study contributes to the evidence base, raters may consider if this affects directness and, if yes, downgrading may be appropriate.

**(4) IMPRECISION**

GRADE Definition. Results are imprecise when studies include relatively few patients and few events and thus have wide confidence intervals around the estimate of the effect. In this case guideline panel will judge the quality of the evidence lower than it otherwise would because of resulting uncertainty in the results (for further details see the GRADEprofiler instructions).

**The following criteria will be followed by WHO raters on IMPRECISION**:

If (a) the overall number of individuals included in trials is low (between 200 and 100 individuals, both treatment arms) or (b) the 95% confidence interval includes both 1) no effect and 2) appreciable benefit or appreciable harm = - 1 (serious imprecision)

If (a) the overall number of individuals included in trials is very low (less than 100 individuals, both treatment arms) and (b) the 95% confidence interval includes both 1) no effect and 2) appreciable benefit or appreciable harm = - 2 (very serious imprecision)

NOTE: For continuous outcomes “no effect” means a SMD with a confidence interval that crosses zero; appreciable benefit or appreciable harm means that the upper or lower confidence limit crosses an effect size of 0.5 in either direction. For dichotomous outcomes “no effect” means an estimate with a confidence interval that crosses one; appreciable benefit or appreciable harm means that the upper or lower confidence limit crosses a risk of 2.0 or 0.5.

*A different criterion may be followed by WHO raters in exceptional situations. Explanation should be reported as footnote in the corresponding GRADE table.*

**(5) REPORTING BIAS**

GRADE Definition. Publication bias is a systematic underestimate or an overestimate of the underlying beneficial or harmful effect due to the selective publication of studies. Publication bias arises when investigators fail to report studies they have undertaken (typically those that show no effect). Methods to detect the possibility of publication bias in systematic reviews exist, although authors of the reviews and guideline panels must often guess about the likelihood of publication bias. A prototypical situation that should elicit suspicion of publication bias occurs when published evidence is limited to a small number of trials, all of which are showing benefits of the studied intervention.

**The following criteria will be followed by WHO raters on REPORTING BIAS**:

If the graphical inspection of the funnel plot suggests some asymmetry, or if any other reasons (to be recorded as footnote) suggest that reporting bias might have had an impact on the overall summary estimate (for example: unpublished grey literature was not included) = - 1

If the graphical inspection of the funnel plot suggests high asymmetry, or if any other reasons (to be recorded as footnote) suggest that reporting bias might have had a high impact on the overall summary estimate (for example: unpublished grey literature was not included) = - 2

*A different criterion may be followed by WHO raters in exceptional situations. Explanation should be reported as footnote in the corresponding GRADE table.*

________________________________________

**Upgrading the evidence according to the GRADE methodology** (<http://www.gradeworkinggroup.org/index.htm>):

**(6) DOSE-RESPONSE GRADIENT**

*PLEASE NOTE: In randomized trials, and in observational studies downgraded for any reason, do not rate the presence of dose-response gradient and choose no. You should assess if there was a dose-response gradient only in observational studies not downgraded for any reason.*

The presence of a dose-response gradient may increase our confidence in the findings of observational studies and thereby increase the quality of evidence. Only observational studies with no threats to validity (not downgraded for any reason) can be upgraded.

**(7) LARGE MAGNITUDE OF EFFECT**

You should assess if the effect was large or very large and, if so, upgrade the quality of evidence accordingly for this outcome. For observational studies, only studies with no important threats to validity (not downgraded for any reasons) should be upgraded.

To rate magnitude of the effect:

- If the effect was not large (RR between 0.5 and 2.0) choose no
- If the effect was large (RR either >2.0 or <0.5 based on consistent evidence from at least 2 studies, with no plausible confounders) choose RR >2 or <0.5
  «this will upgrade the quality of evidence for this outcome by 1 level»
- If the effect was very large (RR either >5.0 or <0.2 based on direct evidence with no major threats to validity) choose RR >5 or <0.2
  «this will upgrade the quality of evidence for this outcome by 2 levels»
- Explain your choice in a footnote whenever you upgrade the quality of evidence for any reason, because it is important for others to understand your choice.

**(8) EFFECT OF ALL PLAUSIBLE CONFOUNDING**

On occasion, all plausible confounding from observational studies or randomized trials may be working to reduce the demonstrated effect or increase the effect if no effect was observed.
For example, if only sicker patients receive an experimental intervention or exposure, yet they still fare better, it is likely that the actual intervention or exposure effect is larger than the data suggest. For observational studies, only studies with no important threats to validity (not downgraded for any reasons) should be upgraded.
